# Rheumatoid nodule masquerading as a bone metastasis: a diagnostic case report

**DOI:** 10.1097/MS9.0000000000003559

**Published:** 2025-07-16

**Authors:** Bal Krishna Subedi, Rafal Ali, Carlos O Pena, Naveen Gautam, Fabian Q Rodriguez

**Affiliations:** a Department of Internal Medicine, Jefferson Einstein Montgomery Hospital (Einstein Medical Center Montgomery), East Norriton, Pennsylvania, USA; b Department of Rheumatology, Jefferson Einstein Philadelphia Hospital (Einstein Medical Center Pennsylvania), Philadelphia, Pennsylvania, USA; c Department of General Medicine, Gulmi Durbar Basic Hospital, Gulmi, Nepal

**Keywords:** anti-CCP antibodies, intraosseous rheumatoid nodule, lytic bone lesion, malignancy mimic, rheumatoid arthritis

## Abstract

**Introduction and importance:**

Rheumatoid arthritis (RA) presents with extra-articular manifestations in 15–25% of cases. Intra-osseous rheumatoid nodules occur in <1% of patients and mimic metastatic disease on imaging. When extra-articular features precede joint symptoms, diagnostic delays are common.

**Case presentation:**

A 78-year-old male smoker presented with chronic cough and dyspnea. CT revealed interstitial lung disease and a pulmonary nodule. PET-CT demonstrated multiple FDG-avid osteolytic lesions in ribs, scapula, and spine (SUV 4.2–8.7), suggesting metastatic malignancy. Initial biopsies showed inflammation without malignancy. Four months later, the patient developed polyarthralgia, morning stiffness, and 8-kg weight loss. Hand radiographs revealed erosive arthropathy. Laboratory tests showed rheumatoid factor 240 IU/mL, anti-CCP 185 U/mL, ESR 68 mm/hr, and CRP 42 mg/L. Repeat scapular biopsy identified necrobiotic granulomas consistent with rheumatoid nodules.

**Clinical discussion:**

Intra-osseous rheumatoid nodules result from immune complex-mediated tissue necrosis. Inflammatory cytokines activate osteoclasts, creating lytic lesions with high metabolic activity indistinguishable from metastases on PET imaging. Extra-articular manifestations precede joint symptoms in 10–15% of RA cases. Anchoring bias toward malignancy in an elderly smoker with pulmonary nodules and osteolytic lesions delayed alternative diagnoses. Negative initial biopsies and subsequent development of erosive arthropathy prompted diagnostic reconsideration.

**Conclusion:**

Extra-articular RA can masquerade as metastatic disease. Intra-osseous rheumatoid nodules represent rare manifestations requiring histological confirmation. Clinicians should consider systemic autoimmune diseases when evaluating unexplained interstitial lung disease with multiple osteolytic lesions, particularly when malignancy screening is negative. Early recognition enables appropriate immunosuppressive therapy and prevents irreversible organ damage.

## Introduction

Rheumatoid arthritis is a systemic autoimmune disease characterized by inflammatory synovitis with broad clinical spectrum^[^1^]^. High-titer rheumatoid factor and anti-citrullinated peptide antibodies define a seropositive phenotype associated with aggressive joint damage and extra-articular manifestations^[^1,2^]^. Significant extra-articular manifestations include interstitial lung disease and rheumatoid nodules^[^3^]^. While interstitial lung disease typically follows articular disease, it represents the initial manifestation in 10–20% of patients, occasionally preceding joint symptoms by years^[^4^]^.

Extra-articular manifestations create substantial diagnostic challenges. Intra-osseous rheumatoid nodules, an exceptionally rare manifestation, appear as lytic, marrow-infiltrating masses on advanced imaging, rendering them radiologically indistinguishable from skeletal metastases^[^5,6^]^. The differential diagnosis for lytic lesions is extensive, but malignancy remains paramount in patients over 40 years^[^7^]^. This diagnostic complexity is amplified by 18 F-fluoro-2-deoxy-D-glucose (FDG) positron emission tomography/CT, as inflammatory conditions demonstrate FDG-avidity and mimic malignancy^[^8^]^. Concurrent findings can anchor diagnostic reasoning toward cancer through cognitive biases, contributing to diagnostic and communication challenges common in rheumatology^[^9-11^]^.

This report presents a complex case of an elderly male smoker-a recognized risk factor for RA-associated interstitial lung disease^[^4^]^-whose presentation was dominated by interstitial lung disease and multiple, progressive, FDG-avid intra-osseous rheumatoid nodules. The case illustrates the diagnostic process required to differentiate this rare rheumatoid arthritis presentation from disseminated malignancy and demonstrates the essential role of histopathology in resolving imaging limitations. This manuscript was prepared in accordance with the SCARE 2025 guidelines^[^12^]^ to ensure accuracy, transparency, and usefulness.

## Case presentation

A 78-year-old man presented with a 2-month history of productive cough with white sputum. He denied dyspnea, chest pain, fever, or systemic symptoms. Medical history included dementia, hyperlipidemia, and 50 pack-year smoking history (quit 30 years prior). He worked 17 years as a sanitary services truck driver with intermittent mask use. Family history revealed maternal intractable cough. He denied connective tissue disease symptoms and took aspirin, atorvastatin, donepezil, and eye drops. Examination revealed stable vital signs, BMI 22 kg/m^2^, left tongue fullness, bilateral fine crackles, and symmetrically diminished breath sounds.

### Initial investigations

**Figure 1.**
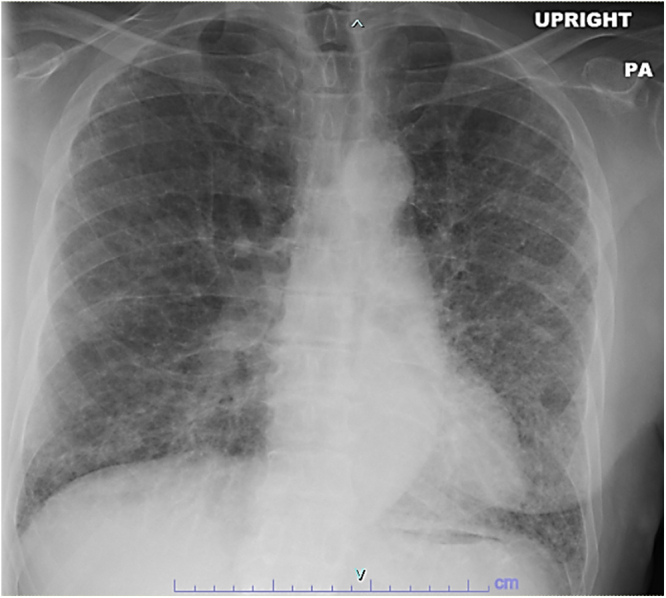
Initial chest X-ray showing diffuse bilateral reticular opacities.

**Figure 2.**
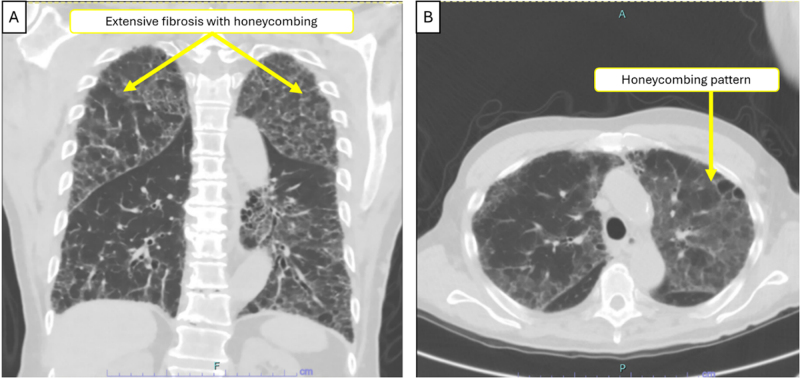
CT chest [frontal (A) and axial sections (B)] showing pre-existing extensive pulmonary fibrosis with honeycombing (arrows).

Chest radiography showed diffuse bilateral reticular opacities (Fig. 1). High-resolution CT demonstrated extensive pulmonary fibrosis with honeycombing, predominantly bilateral lower lobes, consistent with usual interstitial pneumonia pattern (Fig. 2). Additional findings included a 16 × 12 mm irregular left lower lobe nodule highly suspicious for malignancy, bilateral mediastinal lymphadenopathy, left chest wall venous collaterals, and questionable left eighth rib cortical defect. Patient could not tolerate pulmonary function testing. Six-minute walk test showed exertional desaturation to 88%, improving with supplemental oxygen (2 L/min).

### Further investigations and clinical course

Over 24 months, comprehensive evaluation included cryobiopsy, PET-CT, and endobronchial ultrasound. The detailed timeline is summarized in Table 1. PET-CT revealed hypermetabolic activity in the pulmonary nodule and FDG-avid osseous lesions in left eighth rib and right scapula, concerning for metastases. Lymph node sampling and bronchial specimens were negative for malignancy. The patient developed progressive dyspnea and 11.3 kg (25 pounds) weight loss at 6 months.

**HIGHLIGHTS**

- Intra-osseous rheumatoid nodules can present as multiple, destructive, FDG-avid lytic bone lesions, creating a clinical and radiological picture that is nearly indistinguishable from metastatic cancer.
- Severe extra-articular manifestations of rheumatoid arthritis, such as interstitial lung disease and bone lesions, can be the initial presentation of the disease, preceding typical joint symptoms by months.
- Diagnostic delays can be compounded by the non-specificity of FDG-PET/CT scans, as the high metabolic activity in inflammatory rheumatoid nodules mimics malignancy, leading to cognitive errors like anchoring bias.
- Histopathological confirmation is the ultimate arbiter in diagnosing atypical lytic bone lesions; in this case, a definitive diagnosis was only reached after a repeat bone biopsy identified the characteristic necrobiotic palisading granulomas of a rheumatoid nodule.
- Clinicians should consider rheumatoid arthritis in the differential diagnosis for patients with unexplained interstitial lung disease and atypical bone lesions, particularly in older, male smokers with high-titer rheumatoid factor and anti-CCP antibodies.

**Table 1:** Chronological summary of clinical presentation, investigations, and management

Timeframe	Key clinical & diagnostic events	Management & outcomes
Oct–Dec 2022: initial presentation (malignancy mimic)	Presented with a 2-month history of cough. Chest CT revealed ILD (UIP pattern), a suspicious 16 × 12 mm lung nodule, and a lytic lesion in the 8th rib. PET-CT confirmed an FDG-avid 15 mm lung nodule and FDG-avid bone lesions in the rib and right scapula, highly concerning for metastasis. Invasive workup, including bronchoscopy and EBUS, was negative for malignancy.	Patient desaturated to 88% on a 6-minute walk test. Home oxygen therapy at 2 L/min was initiated. Immunosuppressive therapy was deferred due to the high suspicion of malignancy.
Jan–Jul 2023: diagnostic uncertainty & symptom evolution	Patient reported an unintentional weight loss of 25 lbs over 6 months. An initial IR-guided biopsy of the scapular lesion was non-diagnostic, showing a “necrotizing granulomatous reaction” with no malignancy or organisms identified. Arthralgia of the knees and shoulders emerged, which flared significantly after the cessation of a prednisone course. Full ILD serologies were ordered.	Empiric treatment was started: Mycophenolate Mofetil (MMF) 500 mg twice daily. Prednisone 40 mg daily. Nintedanib 100 mg twice daily. Prednisone was tapered to 20 mg daily after 3 weeks of therapy.
Nov 2023–Feb 2024: shift to rheumatologic diagnosis	A 1-year follow-up CT showed progression of multiple lytic bone lesions, with the right scapular lesion now measuring 3.9 cm and a new 1.6 cm lesion in the T8 vertebra. Patient developed overt, symmetric polyarthritis with marked synovitis, ulnar deviation, and early swan-neck deformities. Hand X-rays confirmed erosive arthropathy involving multiple MCP and PIP joints. Key Laboratory Findings: ESR: 85 mm/hr; CRP: 13 mg/L. High-titer RF: 122.6 IU/mL; Anti-CCP: > 250 U/mL. Myeloma workup was negative, with no M-spike identified.	MMF was held due to concern for the progressing lytic bone lesions. A formal rheumatology referral was made.
Mar–Apr 2024: definitive histologic diagnosis	Patient reported new neck pain; a CT of the cervical spine revealed erosions in the dens of C2, consistent with inflammatory arthritis. A repeat, targeted biopsy of the right scapula definitively identified necrobiotic palisading granulomas, confirming the diagnosis of intra-osseous rheumatoid nodules.	Diagnosis of severe, seropositive, erosive RA with extra-articular manifestations was established. A multidisciplinary plan was made to initiate Rituximab therapy.
Jun 2024–Mar 2025: targeted treatment & favorable response	Patient received Rituximab 1000 mg IV infusions on Day 0 and Day 15, followed by repeat doses every 6 months. He responded favorably, with significant improvement in joint symptoms and respiratory status. He became independent of supplemental home oxygen. Follow-up CT imaging showed stable ILD, lung nodules, and lytic bone lesions.	Continued on Rituximab infusions for RA and MMF 500 mg twice daily for ILD. Nintedanib was eventually stopped due to intractable diarrhea.

Due to concerns for malignancy, biopsy of the osseous lesion was planned. Empirical treatment with mycophenolate mofetil 500 mg twice daily, prednisolone 40 mg daily (6 weeks, then tapered), and nintedanib 100 mg twice daily was commenced for ILD. Treatment resulted in symptomatic improvement, increased exercise tolerance, and weight gain.

Cryobiopsy revealed non-specific interstitial pneumonia pattern (mixed cellular and fibrotic) with lymphoplasmacytic bronchitis, eosinophils, interstitial lymphocytic infiltrates, and confirmed fibrosis. IgG4-positive plasma cells were increased but did not meet formal disease criteria. Scapular lesion biopsy showed fibrosing granulation tissue with necrotizing granulomatous reaction, interpreted as autoimmune versus reactive; organism stains were negative.

### Diagnostic re-evaluation

**Figure 3.**
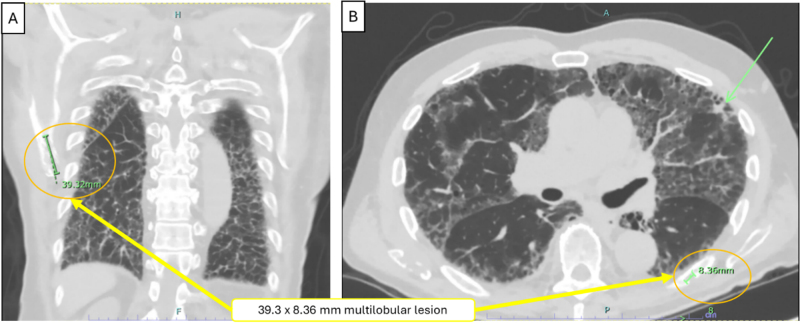
CT chest [frontal (A) and axial view (B)] showing 39.3 × 8.3 mm multilobular lesion in right scapular tip, concerning for neoplastic disease, including multiple myeloma or metastatic carcinoma.

One-year post-presentation, pulmonary function tests showed FEV1/FVC: 0.78, i.e., mild restriction with severely reduced DLCO. Follow-up CT at 15 months showed persistent ILD features (reticulations, septal thickening, ground-glass opacities, traction bronchiectasis, mild honeycombing) interpreted as non-UIP pattern (ILD-RADS 4) with waxing/waning characteristics. The primary left lower lobe nodule remained stable with new smaller left upper lobe nodules and slight increase in another left basal nodule. Lytic bone lesions progressed: right scapular lesion measured 3.9 cm (Fig. 3), left eighth rib lesion enlarged, and new 1.6 cm T8 vertebral lesion developed, concerning for myeloma or metastases. Mycophenolate mofetil was discontinued.

**Figure 4.**
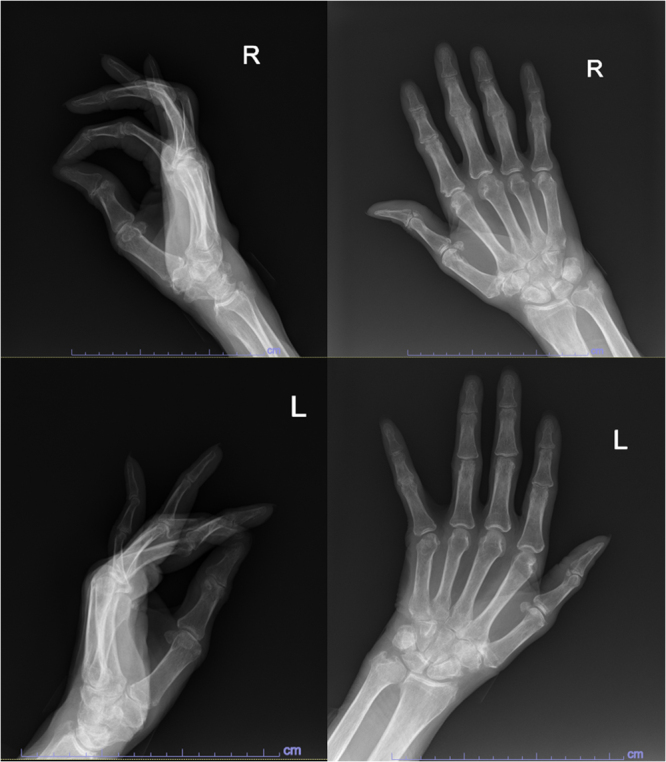
Hand X-ray showing erosive arthropathy involving the ulnar styloid, multiple metacarpophalangeal (MCP), and proximal interphalangeal (PIP) joints.

Rheumatology evaluation for new onset progressively worsening polyarthralgia revealed erosive arthropathy involving ulnar styloid, multiple metacarpophalangeal, and proximal interphalangeal joints on hand radiographs (Fig. 4). Laboratory investigations (Table 2) demonstrated normocytic anemia, hypergammaglobulinemia, hypoalbuminemia, and elevated inflammatory markers (ESR 85 mm/hr, CRP 13 mg/L). Serology showed high-titer rheumatoid factor (122.6 IU/mL) and anti-cyclic citrullinated peptide antibodies (>250 U/mL). Antinuclear antibody remained negative. Serum protein electrophoresis suggested polyclonal gammopathy without M-spike.

**Table 2:** Key laboratory findings and their evolution

Laboratory parameter	Result (initial/follow-up)	Reference range
Inflammatory markers		
C-reactive protein (CRP)	13 (initial)/19 (at 8 months)	1–10 mg/L
Erythrocyte sedimentation rate (ESR)	85 (at rheumatology presentation)	0–30 mm/hr
RA serology		
Rheumatoid factor (RF)	122 (initial)/122.6 (at rheumatology presentation)	<14.0 IU/mL
Anti-CCP antibodies	>250 (initial & at rheumatology presentation)	0–19 units
Hematology		
Hemoglobin	10.5 (at rheumatology presentation)/11.4 (at 8 months)	14.0–18.0 gm/dL
Red blood cell count (RBC)	3.95 (at rheumatology presentation)/3.88 (at 8 months)	4.70–6.10 × 10^6^/mcL
Mean corpuscular volume (MCV)	87.6 (at rheumatology presentation)/94.3 (at 8 months)	81.0–96.0 fL
Serum proteins		
Albumin	2.5 (at rheumatology presentation)/2.7 (at 8 months)	3.9–4.9 gm/dL
Globulin	6.1 (at rheumatology presentation)/6.2 (at 8 months)	1.5–4.5 gm/dL
Total protein	8.6 (at rheumatology presentation)/8.9 (at 8 months)	6.0–8.5 gm/dL
Immunoglobulins		
IgG	3353 (at rheumatology presentation)	603–1613 mg/dL
IgA	896 (at rheumatology presentation)	61–437 mg/dL
IgG subclass-4	449 (at rheumatology presentation)	2–96 mg/dL

**Figure 5.**
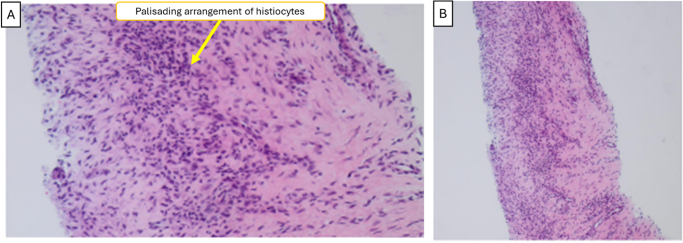
Histopathological examination of scapular lesion biopsy. (A) High-power view showing the characteristic palisading arrangement of histiocytes. (B) Low-power view demonstrating a central area of necrosis (N) surrounded by a rim of palisading histiocytes, consistent with a rheumatoid nodule.

**Figure 6.**
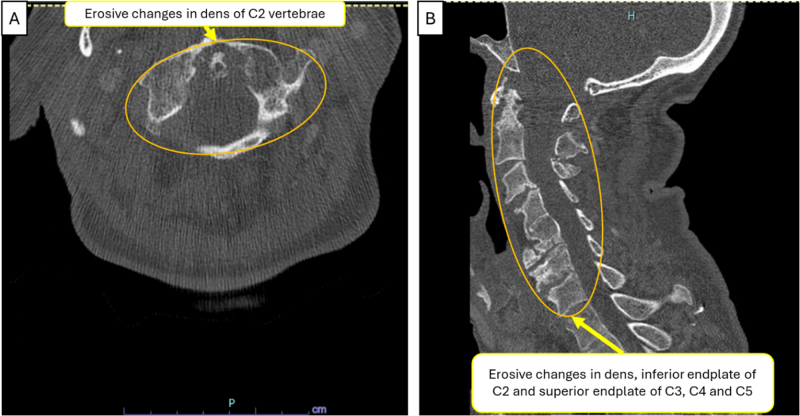
CT cervical spine [axial (A) and sagittal (B)] view showing erosive changes in dens and inferior endplate of C2, extending to superior endplate of C3, C4, and C5, suggestive of inflammatory arthritis

Repeat PET-CT showed decreased uptake in left lower lobe nodule and hilar nodes but persistent bone lesion uptake, interpreted as inflammatory. Repeat right scapular biopsy demonstrated necrobiotic palisading granulomas within chronic inflammation and fibrosis, characteristic of rheumatoid nodules (Fig. 5). Cervical spine CT revealed erosive dens changes consistent with rheumatoid arthritis (Fig. 6). Hematology-oncology excluded myeloma. Antiphospholipid syndrome workup was negative.

### Final diagnosis and management summary

Bilateral symmetric erosive polyarthritis, high-titer rheumatoid factor and anti-cyclic citrullinated peptide positivity, characteristic bone biopsy granulomas, and responsive ILD established seropositive erosive rheumatoid arthritis with extra-articular manifestations: rheumatoid arthritis-associated ILD and atypical intra-osseous rheumatoid nodules mimicking metastases. Treatment was modified to rituximab infusions for rheumatoid arthritis and associated ILD, continued nintedanib, and discontinued mycophenolate mofetil. One-month post-rituximab, activities of daily living improved while respiratory symptoms remained stable. Subsequent imaging showed stable ILD, pulmonary nodule, and bone lesions.

## Discussion

This case demonstrates a complex diagnostic challenge where initial presentation mimicked metastatic lung cancer. The combination of chronic dry cough, suspicious pulmonary nodule with mediastinal lymphadenopathy, and multiple FDG-avid lytic bone lesions in an elderly ex-smoker created diagnostic momentum toward malignancy. The subsequent diagnosis of seropositive rheumatoid arthritis with two major extra-articular manifestations: RA-associated interstitial lung disease and intra-osseous rheumatoid nodulosis demonstrates the heterogeneous clinical presentation of this systemic autoimmune disorder.

The presence of multiple intra-osseous rheumatoid nodules represents the pivotal feature. This manifestation is exceptionally rare, with imaging characteristics closely simulating malignancy^[^5,6^]^. On CT and MRI, these nodules appear as destructive, marrow-replacing lesions with wide, ill-defined transition zones and cortical breakthrough^[^5,7,9^]^. This aggressive appearance, combined with high metabolic activity on PET-CT, renders them nearly indistinguishable from skeletal metastases or myeloma without tissue sampling^[^7,9^]^. Our case aligns with this deceptive radiologic profile, which carries high malignancy predictive likelihood^[^9^]^.

The destructive potential of rheumatoid inflammation is not limited to intra-osseous nodules. Similar diagnostic challenges arise from RA-driven lytic lesions in other atypical skeletal sites, such as the large, malignancy-simulating lesions of the femoral neck described by Gerster *et al*^[^13^]^ or the destructive vertebral lesions mimicking infection reported by Lorber *et al*^[^14^]^. This potential for mimicry extends beyond the skeletal system to visceral organs, including, exceptionally, the central nervous system, where intracranial rheumatoid nodules have been reported to simulate glioblastoma multiforme on imaging^[^15^]^. Reinforcing this point, Brossard-Barbosa *et al* described a similar case of an intracranial nodule presenting as a mass lesion that caused recurrent neurological deficits, which improved only after biopsy-led diagnosis and targeted therapy^[^16^]^. Furthermore, the presentation can mimic infection; Mine *et al* reported a giant subcutaneous nodule presenting with pain, redness, and heat suggestive of a suppurative abscess, where the diagnosis of a rheumatoid nodule was only made on histology^[^17^]^. These cases, along with reports of intra-articular nodules causing mechanical symptoms^[^18^]^, underscore that aggressive rheumatoid disease can invade various tissues and highlight the indispensable role of histopathology in differentiating these rare presentations from their mimics.

The differential diagnosis for multiple osteolytic lesions in elderly patients requires consideration of highest-risk possibilities. Metastatic carcinoma and multiple myeloma remain primary considerations^[^7^]^. Primary bone sarcomas were also included in the differential diagnosis but were deemed improbable. Ewing sarcoma was considered highly unlikely given the patient’s advanced age. While osteosarcoma is known to have a bimodal age distribution that includes older adults, it was also considered less likely. This is because osteosarcoma typically forms osteoblastic or mixed lytic-blastic lesions, which contradicts the purely destructive (lytic) pattern observed in this case^[^7^]^. Eosinophilic granuloma was similarly improbable due to young age predilection. Among non-malignant mimics, chronic osteomyelitis represents a key differential, causing lytic destruction and FDG-avidity^[^8^]^. However, multifocal widespread distribution without clear infection source and repeated negative organism isolation made this unlikely. Osseous sarcoidosis was considered but deemed improbable due to absent systemic features and distinct histopathology. Rheumatoid nodules demonstrate necrotizing, palisading granulomas pathologically distinct from classic non-caseating sarcoidosis granulomas^[^8^]^. IgG4-related disease was excluded by atypical presentation with multifocal destructive bone disease and insufficient IgG4-positive plasma cells.

This malignancy mimicry contributed to diagnostic delay, a multifactorial problem documented in rheumatic diseases^[^10^]^. The prolonged diagnostic course reflects cognitive challenges influenced by anchoring bias, where clinicians rely excessively on initial information for subsequent judgments^[^11^]^. Initial findings in an elderly smoker strongly suggested malignancy, impeding pivot to rare rheumatologic diagnosis despite contrary evidence. Such cognitive biases contribute to diagnostic errors and adverse outcomes^[^11^]^. Diagnostic uncertainty was compounded by challenges in differentiating disease-driven pathology from other processes, analogous to diagnosing DRESS in patients with underlying systemic inflammatory illnesses^[^19^]^. Effective physician-patient communication proves crucial in navigating diagnostic uncertainty. Failure to reach diagnosis despite extensive investigation can erode trust and create communication barriers, compounded by inadequate verification of patient comprehension in routine practice^[^20,21^]^.

Initial negative biopsies did not refute malignancy but prompted prolonged search for elusive primary. Only after overt clinical arthritis development and highly positive serologies did the diagnostic paradigm shift toward rheumatoid arthritis, culminating in definitive histologic diagnosis. This confirms that histopathology remains the ultimate arbiter for atypical, progressive lytic bone lesions^[^8,9^]^.

Concurrent interstitial lung disease further complicated the clinical picture. Extra-articular manifestations can represent initial rheumatoid arthritis presentation, preceding joint symptoms by months or years^[^4,22^]^. This patient’s profile-older age, male sex, smoking history, and high-titer RF and anti-CCP antibodies-aligns with known RA-ILD risk factors^[^2,4^]^. The usual interstitial pneumonia pattern on HRCT carries significant prognostic importance, as this subtype associates with higher mortality^[^2^]^. Persistently elevated inflammatory markers are consistent with RA-ILD cohorts, suggesting higher systemic inflammatory burden^[^2^]^. While this patient presented with severe, destructive extra-articular manifestations, “rheumatoid nodulosis” represents the opposite spectrum-a rare variant in middle-aged men with multiple subcutaneous nodules and cystic bone lesions but minimal systemic inflammation^[^23^]^. Our patient’s aggressive presentation stands in contrast to this more benign variant, highlighting the marked heterogeneity of rheumatoid disease.

## Conclusion

This case demonstrates the diagnostic complexity when rheumatoid arthritis extra-articular manifestations precede joint involvement. Intra-osseous rheumatoid nodules create malignancy mimicry, while FDG-PET/CT non-specificity in differentiating inflammatory from neoplastic processes contributes to diagnostic delays. These delays result from complex referral pathways, cognitive biases including anchoring, and difficulties communicating clinical uncertainty. Risk factors including male sex, smoking history, and high-titer autoantibodies should prompt clinical suspicion for RA-associated interstitial lung disease, with usual interstitial pneumonia pattern indicating poor prognosis. Rheumatoid arthritis should also be considered in unexplained interstitial lung disease with atypical lytic bone lesions, emphasizing the essential role of histopathologic confirmation for accurate diagnosis and management.

## Data Availability

Data sharing is not applicable to this article as no datasets were generated or analyzed during the current study.

## Research registration unique identifying number (UIN)

Research registration is not applicable in this case report.

## Declaration

In accordance with the SCARE 2025 guidelines, the authors declare the use of a generative artificial intelligence (AI) language model in the preparation of this manuscript. The AI tool, ChatGPT (OpenAI, San Francisco, CA), was utilized exclusively for the purpose of improving grammar, spelling, and language clarity. The authors reviewed all AI-generated suggestions and retained full responsibility for the scientific integrity, accuracy, and interpretation of the final manuscript content. No AI was used for data analysis, image generation, or drawing scientific conclusions.

## Patient perspective

The patient endured a prolonged diagnostic journey starting with a persistent cough. Over months, he experienced distressing symptoms including worsening shortness of breath on exertion, significant weight loss, and noticeable exercise limitations requiring oxygen. While initial treatments provided some relief in symptoms and weight regain, they also introduced side effects like hypertension. He later developed joint pain that fluctuated with medication changes. Ultimately, after the correct diagnosis of rheumatoid arthritis was made and targeted treatment began, he reported a tangible improvement in his ability to manage daily activities, although his breathing difficulties persisted to some extent, necessitating continued intermittent oxygen use.
